# A Dominant Heterozygous Mutation in *COG4* Causes Saul–Wilson Syndrome, a Primordial Dwarfism, and Disrupts Zebrafish Development via Wnt Signaling

**DOI:** 10.3389/fcell.2021.720688

**Published:** 2021-09-14

**Authors:** Zhi-Jie Xia, Xin-Xin I. Zeng, Mitali Tambe, Bobby G. Ng, P. Duc S. Dong, Hudson H. Freeze

**Affiliations:** ^1^Human Genetics Program, Sanford Burnham Prebys Medical Discovery Institute, La Jolla, CA, United States; ^2^Development, Aging and Regeneration Program, Sanford Burnham Prebys Medical Discovery Institute, La Jolla, CA, United States; ^3^National Centre for Advancing Translational Sciences, National Institutes of Health, Bethesda, MD, United States; ^4^Graduate School of Biomedical Sciences, Sanford Burnham Prebys Medical Discovery Institute, La Jolla, CA, United States

**Keywords:** Saul–Wilson syndrome, COG4, glypican, WNT4, zebrafish, early development

## Abstract

Saul–Wilson syndrome (SWS) is a rare, skeletal dysplasia with progeroid appearance and primordial dwarfism. It is caused by a heterozygous, dominant variant (p.G516R) in COG4, a subunit of the conserved oligomeric Golgi (COG) complex involved in intracellular vesicular transport. Our previous work has shown the intracellular disturbances caused by this mutation; however, the pathological mechanism of SWS needs further investigation. We sought to understand the molecular mechanism of specific aspects of the SWS phenotype by analyzing SWS-derived fibroblasts and zebrafish embryos expressing this dominant variant. SWS fibroblasts accumulate glypicans, a group of heparan sulfate proteoglycans (HSPGs) critical for growth and bone development through multiple signaling pathways. Consistently, we find that glypicans are increased in zebrafish embryos expressing the *COG4^*p*.G516R^* variant. These animals show phenotypes consistent with convergent extension (CE) defects during gastrulation, shortened body length, and malformed jaw cartilage chondrocyte intercalation at larval stages. Since non-canonical Wnt signaling was shown in zebrafish to be related to the regulation of these processes by glypican 4, we assessed *wnt* levels and found a selective increase of *wnt4* transcripts in the presence of *COG4^*p*.G516R^*. Moreover, overexpression of *wnt4* mRNA phenocopies these developmental defects. LGK974, an inhibitor of Wnt signaling, corrects the shortened body length at low concentrations but amplifies it at slightly higher concentrations. WNT4 and the non-canonical Wnt signaling component phospho-JNK are also elevated in cultured SWS-derived fibroblasts. Similar results from SWS cell lines and zebrafish point to altered non-canonical Wnt signaling as one possible mechanism underlying SWS pathology.

## Introduction

Saul–Wilson syndrome (SWS) is a rare skeletal dysplasia characterized by profound short stature and distinctive craniofacial features such as prominent forehead, prominent eyes, and micrognathia (Saul and Wilson, 1990; Ferreira et al., 2020). Recently, we defined a specific heterozygous COG4 substitution (p.G516R) as the molecular basis of this rare form of primordial dwarfism (Ferreira et al., 2018). COG4 is one of the eight subunits of the conserved oligomeric Golgi (COG) complex regulating protein trafficking and Golgi homeostasis (Ungar et al., 2002). Biallelic pathogenic variants in *COG4* and other COG subunits cause multiple human congenital disorders of glycosylation (CDGs) (D’Souza et al., 2020). COG4-CDG individuals have a very severe, usually lethal, phenotype with dysmorphia, neurological and intellectual disabilities, and altered N-glycosylation with an almost total loss of COG4 (Reynders et al., 2009; Ng et al., 2011). However, SWS subjects show very different features, since their N-glycans and their intellectual and neurological features appear normal (Ferreira et al., 2018). At the cellular level, the *COG4^*p*.G516R^* variant disrupted protein trafficking by accelerating brefeldin-A (BFA)-induced retrograde transport and delaying anterograde transport, causing the collapse of the Golgi stacks. This interrupted bidirectional trafficking between the ER and the Golgi and altered decorin, a proteoglycan (Ferreira et al., 2018), indicate that modified proteoglycans may be involved in the pathogenesis of SWS.

Proteoglycans play critical roles in multiple cell processes at the cellular, tissue, and organismal levels, and their deficiencies cause bone and connective tissue disorders (Iozzo and Schaefer, 2015; Paganini et al., 2019). Several proteoglycan deficiencies have been studied in zebrafish, a powerful vertebrate model for studying CDGs and skeletal disorders, with some showing a shortened body axis (Zoeller et al., 2009; Cline et al., 2012; Lee et al., 2020; Tonelli et al., 2020; Dietrich et al., 2021). Besides a body axis defect, morpholino-mediated knockdown of zebrafish decorin (*dcn*) displayed relatively severe defects in body curvature associated with a curved or not fully extended tail (Zoeller et al., 2009). Defects in glypicans, a group of heparan sulfate proteoglycans (HSPGs), can cause abnormal skull and skeletal dysplasia in both humans (Simpson–Golabi–Behmel syndrome: glypican 3, GPC3; Keipert syndrome: glypican 4, GPC4) and zebrafish (Knypek: Gpc4) (LeClair et al., 2009; Tenorio et al., 2014). Interestingly, studies of *knypek* (*kny*/*gpc4*) mutant zebrafish demonstrated that Gpc4 deficiency causes chondrocyte stacking and intercalation defects in Meckel’s cartilage, which is not seen in *dcn* morphants or *chondroitin sulfate proteoglycan 4* (*cspg4*)-deficient zebrafish (Topczewski et al., 2001; Zoeller et al., 2009; Hu et al., 2012; Lee et al., 2020). *kny* adult zebrafish also show craniofacial defects including a smaller head, domed skull, and shorter jawbones, reminiscent of some of the clinical features of SWS individuals (Topczewski et al., 2001; LeClair et al., 2009). Studies also found that optimized expression of *gpc4* can suppress the defects caused by Wnt11f2 (formerly known as Wnt11/Silberblick; Postlethwait et al., 2019) deficiency, indicating the role of Gpc4 in the Wnt signaling pathway, probably as a Wnt coreceptor (LeClair et al., 2009). It is worth noting that both absence and high expression of *gpc4* lead to the loss of ability to suppress Wnt11f2 deficiency (Topczewski et al., 2001). Presumably, abnormalities arise when either these receptors or ligands are outside the optimal ratio. Taken together, the similarity between *kny* mutant zebrafish craniofacial defects and some of the SWS individuals’ clinical features encouraged us to use zebrafish as a vertebrate model to explore the underlying pathological mechanism of SWS.

The zebrafish Cog4 is 72% identical to human COG4, and the amino acid corresponding to the SWS mutation site is conserved across multiple vertebrate species.^1^ Zebrafish that lack the Cog4 protein show phenotypes consistent with the clinical features of COG4-CDG individuals, including defective synthesis of N- and O-linked glycans and decreased glycosphingolipid complexity (Clement et al., 2019). SWS individuals show very different features compared to COG4-CDG individuals, and SWS cells show accelerated BFA-induced retrograde trafficking in contrast to COG4-CDG cells. Considering these facts, a zebrafish model for the SWS-specific variant is highly desired to investigate phenotypic features and the possible pathogenesis of this heterozygous mutation in *COG4*.

In this study, we utilize SWS-derived fibroblasts and a zebrafish system to test a specific heterozygous COG4 substitution (p.G516R), which is causal for SWS. We assessed a broader category of proteoglycans and found a consistent increase of glypican level in SWS-derived fibroblasts and zebrafish expressing human *COG4^*p*.G516R^* variant. Further studies on non-canonical Wnts revealed that the presence of *COG4^*p*.G516R^* specifically elevated the *wnt4* transcript, but not *wnt5a*, *wnt5b*, or *wnt11f2*. Overexpression of *wnt4* phenocopies the *COG4^*p*.G516R^* zebrafish, and the Wnt inhibitor LGK974 suppresses the defects caused by the expression of *COG4^*p*.G516R^*. These findings suggest that disrupted Wnt signaling is one possible mechanism underlying the pathogenesis of SWS.

## Results

### Fibroblasts From SWS Individuals Accumulate Glypicans on the Cell Surface

**FIGURE 1:**
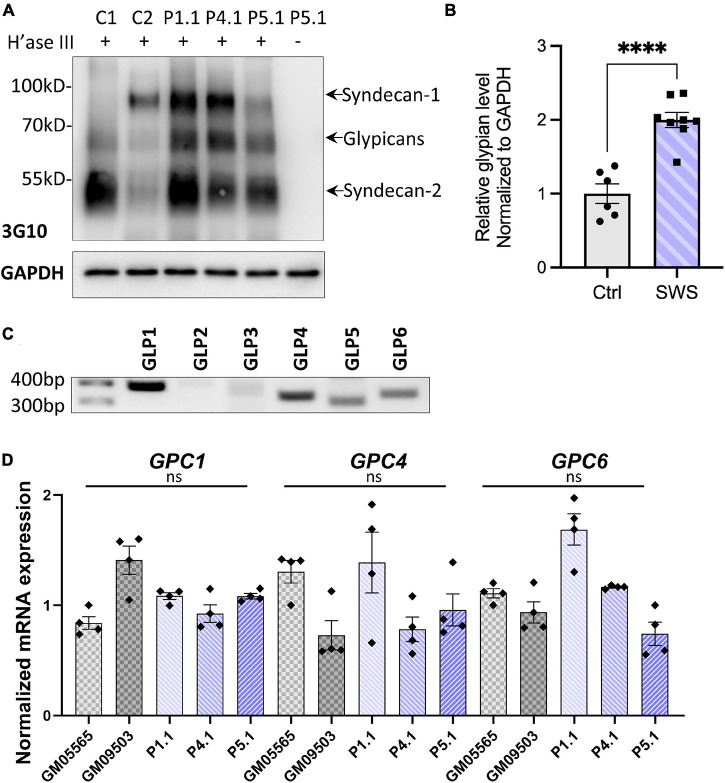
SWS-derived fibroblasts show altered HSPGs and glypicans after heparinase III (H’ase III) digestion. **(A)** Western blotting of ΔHS-stub using 3G10 antibody following heparinase III digestion of three SWS-derived fibroblasts and two control fibroblasts. C1 and C2 are control fibroblasts. C1, GM08429; C2, GM08680. P1.1, P4.1, and P5.1 are SWS-derived fibroblasts. **(B)** Quantitation assay of glypican band density in **(A)** and two other replicates. The data are presented as mean ± SEM. An unpaired two-tailed *t*-test was used. ^*⁣*⁣**^p < 0.0001. **(C)** Agarose gel of six human glypicans after reverse-transcript PCR. **(D)** qPCR of three dominant glypicans in SWS-derived fibroblasts. The relative glypican level was normalized to GAPDH. The graphs represent the 2^–ΔΔCt^ values. Experiments were performed in triplicates with similar results.

As one of the major components of extracellular matrix (ECM) and cell membrane proteins, proteoglycans comprise a large, heterogeneous group including HSPGs and chondroitin sulfate proteoglycans (CSPGs). Decorin is a predominant proteoglycan in human skin, covalently linked with one glycosaminoglycan chain (GAG), which requires normal function of the Golgi for its posttranslational modification. Our results showing abnormal decorin modification encouraged us to study other proteoglycans in SWS cells. As a first step in determining which proteoglycan may change, we analyzed the core proteins of HSPGs using an antibody (3G10) against HS-stubs (a heparan sulfate neo-epitope) that appear only after heparinase III digestion. As shown in Figure 1A, glypicans, syndecan 1, and syndecan 2 were the most prevalent cell surface HSPGs in dermal fibroblasts. Compared to syndecans, glypicans showed consistent increases in all three SWS cell lines with an average twofold elevation (Figure 1B). We performed qPCR to assess whether transcript abundance is also altered. There are six glypicans in humans, and among those, glypicans 1, 4, and 6 are present in multiple tissues, while glypicans 3 and 5 are restricted to the ovary and brain. Glypican 2 is specifically expressed in the nervous system during embryonic development (Thul and Lindskog, 2018; Guo et al., 2020). As seen in Figure 1C, glypicans 1, 4, 5, and 6 were detectable in dermal fibroblasts; however, we did not observe a significant change in any of their transcript abundance (Figure 1D). We hypothesize that this increase of glypicans results from SWS COG4-dependent abnormal trafficking or turnover, instead of transcriptional regulation. A similar strategy was applied to study CSPGs. Chondroitinase ABC was used to digest CSPGs followed by immunoblotting against the ΔDi-6S. There was a general decrease of CSPG core proteins in three out of four SWS-derived cell lines (Supplementary Figure 1). Considering that glypicans show the most prominent difference between controls and SWS-derived cells and the phenotypes of *gpc4* mutant zebrafish, we focused on glypicans in our study.

### Expression of Human *COG4*^*p*.G516R^ in Zebrafish Increases Glypican Proteins

**FIGURE 2:**
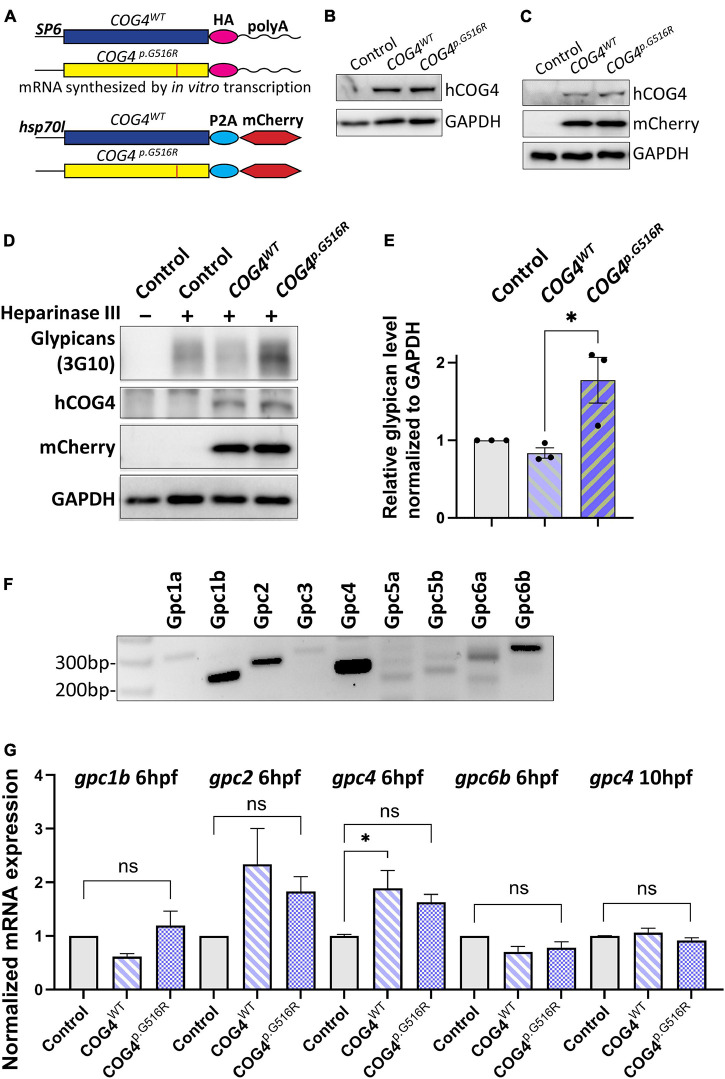
Expression of human *COG4^*p*.G516R^* in zebrafish increases the protein level of glypicans. **(A–C)** Expression of human *COG4^*WT*^* and *COG4^*p*.G516R^* in zebrafish after mRNA or DNA injection. **(A)** The scheme of *COG4* constructs for *in vitro* transcription (top) and DNA injection (bottom). **(B)** Western blot at 24 hpf to detect the presence of COG4 after mRNA injection. **(C)** Western blot at 48 hpf to detect COG4 after DNA injection; heat shock was performed at 24 hpf for 2 h at 38°C. **(D–G)** Glypican analysis in zebrafish. **(D)** Western blotting of ΔHS-stub using 3G10 antibody following heparinase III digestion of control and embryos injected with *COG4^*WT*^* or *COG4^*p*.G516R^* mRNA at 3 dpf. **(E)** Quantitation assay of glypican band density in **(D)** and two more replicates. The data are presented as mean ± SEM. An unpaired two-tailed *t*-test was used. ^∗^*p* < 0.05. **(F)** mRNA expression of glypican genes by RT-PCR using cDNA from control embryos at 6 hpf. **(G)** qPCR analyses of highly expressed glypicans in control and zebrafish embryos injected with human *COG4^*WT*^* or *COG4^*p*.G516R^* mRNA. The relative glypican level was normalized to β*-actin*. The graphs represent the 2^–ΔΔCt^ values. One-way ANOVA with Tukey’s multiple comparison tests was applied. ns, not significant; ^∗^*p* < 0.05. Experiments were performed in triplicates with similar results.

To study the impact of the SWS variant on skeletal development, we used zebrafish as a vertebrate model. Since SWS is a dominant disorder, we overexpressed the human SWS allele in developing zebrafish embryos. Human *COG4^*WT*^* and *COG4^*p*.G516R^* mRNAs or DNA constructs, as shown in Figure 2A, were injected into one-cell-stage embryos. The presence of human COG4 in zebrafish was confirmed by an antibody specifically recognizing human COG4 protein (Figures 2B,C). Lacking a zebrafish Cog4 antibody makes it impossible to determine the relative expression level of *COG4^*p*.G516R^*; therefore, we include *COG4^*WT*^* as an injected control to ensure comparable expression of *COG4^*p*.G516R^* in zebrafish. Both *COG4^*WT*^* and *COG4^*p*.G516R^* were expressed at a very similar level (Figures 2B,C), and no adverse effects were seen in embryos expressing *COG4^*WT*^* compared to uninjected siblings.

We first checked glypican proteins using the same strategy as in SWS-derived cells. Interestingly, we found increased glypicans in embryos expressing *COG4^*p*.G516R^* but not *COG4^*WT*^* at 3 dpf (days post-fertilization) (Figures 2D,E), consistent with the observation in SWS-derived fibroblasts. Zebrafish have 10

glypicans expressed at different developmental stages (Gupta and Brand, 2013). At 6 hpf (hours post-fertilization), we detected five glypicans, namely, Gpc1b, Gpc2, Gpc4, Gpc6a, and Gpc6b, by RT-PCR (Figure 2F), followed by qPCR to compare their transcript levels. We found that the *gpc2* and *gpc4* transcript levels are elevated in both *COG4^*WT*^* and *COG4^*p*.G516R^* embryos compared to the uninjected control, but there is no significant difference between *COG4^*WT*^* and *COG4^*p*.G516R^* (Figure 2G). At 10 hpf, the transcript level of endogenous *gpc4* fell to a level comparable to that of the control, with no distinction between *COG4^*WT*^* and *COG4^*p*.G516R^* (Figure 2G). The *gpc2* mRNA level at 10 hpf was barely detectable.

### Expression of Human *COG4*^*p*.G516R^ in Zebrafish Shortens Body Length and Causes Abnormal Chondrocyte Stacking and Intercalation

**FIGURE 3:**
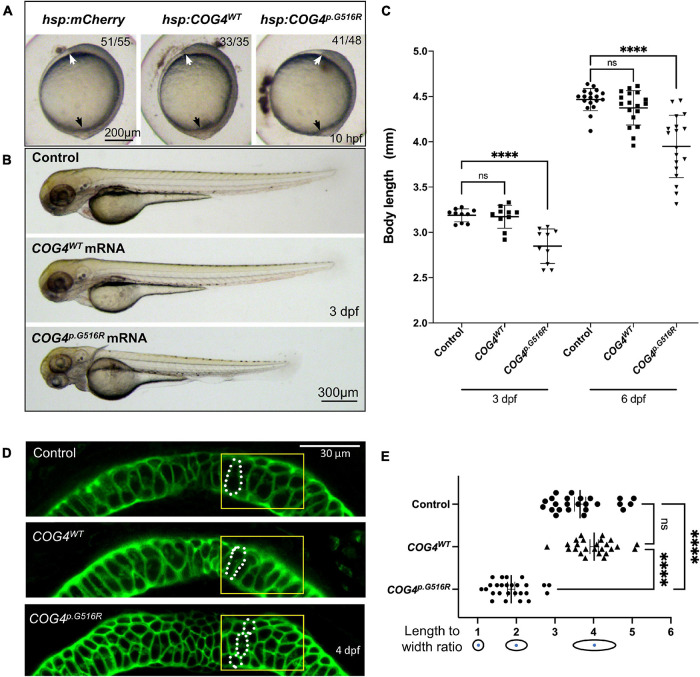
Expression of human *COG4^*p*.G516R^* impairs zebrafish early development and chondrocyte intercalation. **(A,B)** Expression of *COG4^*p*^.^*G*516R^* in zebrafish causes gastrulation defects and shortened body axis. **(A)** Lateral view of representative embryos. Anterior to the top. Compared to the control construct *hsp70l:mCherry* and *COG4^*WT*^*, embryos expressing *COG4^*p*.G516R^* show an axis extension defect at 10 hpf. White arrow points to the head region, and the black arrow points to the tailbud. Expression of *COG4^*p*.G516R^* mRNA causes similar results. **(C)** Graphs show the measured body length of each group at 3 and 6 dpf. The data are presented as mean ± SD. One-way ANOVA with Tukey’s multiple comparison tests was applied. ^****^p < 0.0001; ns, not significant. **(D)** Expression of *COG4^*p*.G516G^* causes craniofacial abnormalities. Ventral view of representative Meckel’s cartilage of zebrafish larvae at 4 dpf after WGA staining and imaged by a confocal microscope. Dotted circular lines highlight chondrocyte cell shape and their relative configuration with each other. **(E)** Graphical representation of the length-to-width ratio of chondrocytes in the region of interest, yellow box in **(D)**. Individual cell length-to-width ratio was measured in three representative Meckel’s cartilage images of each group. The data are presented as mean ± SEM. One-way ANOVA with Tukey’s multiple comparison tests was applied. ^****^p < 0.0001; ns, not significant. Experiments were performed in triplicates with similar results.

We examined zebrafish body length at different stages to assess the developmental phenotypes caused by *COG4^*p*.G516R^* expression. At the end of gastrulation, embryos expressing the *COG4^*p*.G516R^* variant exhibit shorter axis extension (Figure 3A), suggesting an abnormal convergent extension (CE) movement (Topczewski et al., 2001). Embryos expressing *COG4^*WT*^* developed normally (Figure 3A). We tracked these injected embryos at later stages and found that expression of *COG4^*p*.G516R^* causes a shortened anterior–posterior (AP) body axis (Figure 3B) by an average of 18% at 3 dpf and 10% at 6 dpf (Figure 3C).

To investigate whether the presence of COG4^*p*.G516R^ in zebrafish impacts chondrocyte development, zebrafish at 4 dpf were stained with wheat germ agglutinin (WGA), a lectin binding to glycoproteins in cartilage ECM to visualize chondrocyte morphology. In embryos expressing *COG4^*p*.G516R^*, Meckel’s cartilage was deformed, shown as defects in chondrocyte stacking and elongation (Figure 3D). Compared to *COG4^*WT*^* embryos, more rounded chondrocytes were present in Meckel’s cartilage (Figure 3E). These defects can be observed as late as 7 dpf, as shown by Alcian blue staining (Supplementary Figure 2). Chondrocyte stacking and intercalation problems further confirmed that abnormal glypican levels could be one of the pathogenetic mechanisms involved in SWS. Other phenotypes seen in *COG4^*p*.G516R^*-injected embryos also include abnormal, stunted fin, and cyclopia (Supplementary Figures 3A,B).

### Expression of Human *COG4*^*p*.G516R^ Elevates the *wnt4* Transcript in Zebrafish

**FIGURE 4:**
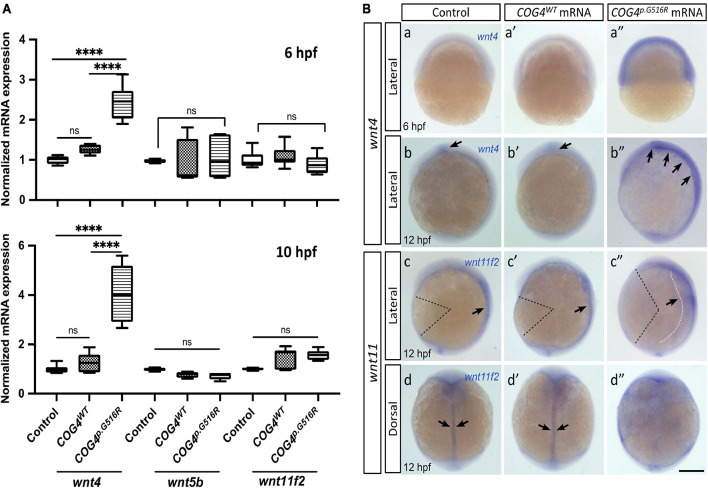
Expression of human *COG4^*p*.G516R^* elevates the *wnt4* transcript in zebrafish embryos. **(A)** Quantitative PCR analyses of selective non-canonical Wnt pathway ligands in zebrafish embryos injected with human *COG4^*WT*^* or *COG4^*p*.G516R^* mRNA. At 6 hpf, *wnt4* expression is significantly upregulated in embryos injected with *COG4^*p*.G516R^* mRNA, but not in those injected with *COG4^*WT*^* mRNA. Similar upregulation of *wnt4* transcripts is observed at 10 hpf as well. Bar graphs represent average gene expression relative to the housekeeping gene β*-actin*. The data are presented as mean ± SD. One-way ANOVA with Tukey’s multiple comparison tests was used. ^****^p < 0.0001; ns, not significant. **(B)** Representative images of whole-mount *in situ* hybridization from control and treated embryos. Whole-mount *in situ* hybridization analyses demonstrate that *wnt4* expression is elevated in embryos injected with *COG4^*p*.G516R^* mRNA (a″, at 6 hpf; b″ at 12 hpf), but there are no obvious changes in embryos injected with human *COG4^*WT*^* mRNA (a′ and b′). (b, b’) Arrows point to the restricted *wnt4* expression domain in the hindbrain of zebrafish embryos at 12 hpf; (b″) arrows point to expanded expression of *wnt4* in the hindbrain region. At 12 hpf, *wnt11f2* expression intensity is not significantly changed in embryos injected with *COG4^*p*.G516R^* mRNA (c″, d″), compared to either control siblings (c, d) or human *COG4^*WT*^* mRNA-injected embryos (c′, d′). The dotted black color lines landmark the degree of angle between the head and tail, which is much more increased in embryos injected with *COG4^*p*.G516R^* mRNA, suggesting defects in extension movement during gastrulation. Arrows in c, c′ and d, d″ indicate that *wnt11f2* is restricted to the dorsal midline; however, its expression pattern is dispersed in *COG4^*p*.G516R^* mRNA-injected embryos, suggesting that the convergence movement is impaired (c″, white dotted line and arrow; d″, there is no clear midline expression). Scale bars: 200 μm. Experiments were performed in triplicates with similar results.

Glypican 4 plays an essential role in gastrulation movements and contributes to craniofacial morphogenesis probably through planar cell polarity (PCP)/non-canonical Wnt signaling (Topczewski et al., 2001; Sisson et al., 2015). Therefore, we assessed the expression of non-canonical *wnt*s by qPCR and their spatial–temporal transcription pattern by whole-mount *in situ* hybridization. We found that *COG4^*p*.G516R^* mRNA-injected embryos contain more *wnt4* compared to controls (Figure 4A). In contrast, no significant changes were detected in a few other non-canonical *wnt*s, such as *wnt5b* and *wnt11f2*. Also, the upregulation of *wnt4* shows a dose-dependent response to the amount of *COG4^*p*.G516R^* mRNA injected (Supplementary Figure 4). This elevated transcript level of *wnt4* was further confirmed by whole-mount *in situ* hybridization (Figure 4B). At 6 hpf, *wnt4* was not detectable in either control or *COG4^*WT*^* mRNA-injected embryos, but it was in *COG4^*p*.G516R^*-mRNA injected embryos. At 10 hpf, *wnt4* is confined to the hindbrain in control and *COG4^*WT*^* embryos, but *COG4^*p*.G516R^* embryos had both increased and spatially expanded expression of *wnt4.* The *wnt11f2* transcript level did not change, but the distribution pattern was significantly altered. In uninjected control and *COG4^*WT*^* embryos, *wnt11f2* was restricted to the dorsal midline. However, its expression pattern is dispersed in *COG4^*p*.G516R^* embryos. No measurable difference was found for β-catenin protein, a marker of canonical Wnt signaling between *COG4^*p*.G516R^* and controls (Supplementary Figure 6).

### Overexpression of Zebrafish *wnt4* Causes Shortened Body Length and Malformed Meckel’s Cartilage

**FIGURE 5:**
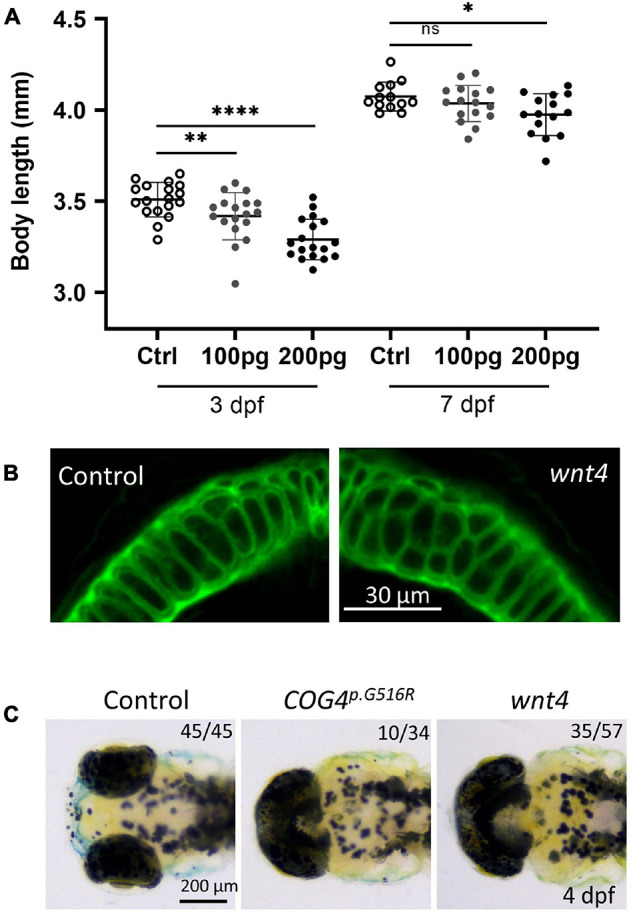
Overexpression of *wnt4* phenocopies zebrafish embryos injected with the *COG4^*p*.G516G^* mRNA. **(A)** Graphs show the measured body length of each group after *wnt4* mRNA injection at 3 and 7 dpf. The data are presented as mean ± SD. An unpaired two-tailed *t*-test was used. ^****^p < 0.0001; ^∗∗^*p* < 0.01; ^∗^*p* < 0.05; ns, not significant. **(B)** Overexpression of *wnt4* causes abnormal chondrocyte stacking and intercalation at Meckel’s cartilage. Ventral view of representative Meckel’s cartilage of zebrafish larvae after *wnt4* injection at 4 dpf following WGA staining and imaged by a confocal microscope. **(C)** Overexpression of *wnt4* causes cyclopia as expression of the *COG4^*p*.G516G^* variant. Dorsal view of representative images of cyclopia compared to control. Two hundred picograms of *wnt4* or *COG4^*p*.G516G^* mRNA was used per embryo. Experiments were performed in triplicates with similar results.

In zebrafish, *wnt4* overexpression has been studied at earlier embryonic stages and was found to inhibit cell movements without altering cell fates (Ungar et al., 1995; Topczewski et al., 2001). Thus, we evaluated the phenotypes of *wnt4* overexpression at a later embryonic stage. At 4 dpf, *wnt4* mRNA-injected embryos showed shortened body length in response to the dosage of *wnt4* injected (Figure 5A). Interestingly, we also found abnormal chondrocyte stacking in Meckel’s cartilage (Figure 5B) and cyclopia (Figure 5C). These data show that overexpression of *wnt4* phenocopies shortened body length and malformed chondrocyte intercalation in *COG4^*p*.G516R^*-injected zebrafish.

### WNT Inhibitor LGK974 Suppresses the Defects Caused by *COG4*^*p*.G516R^ Expression

**FIGURE 6:**
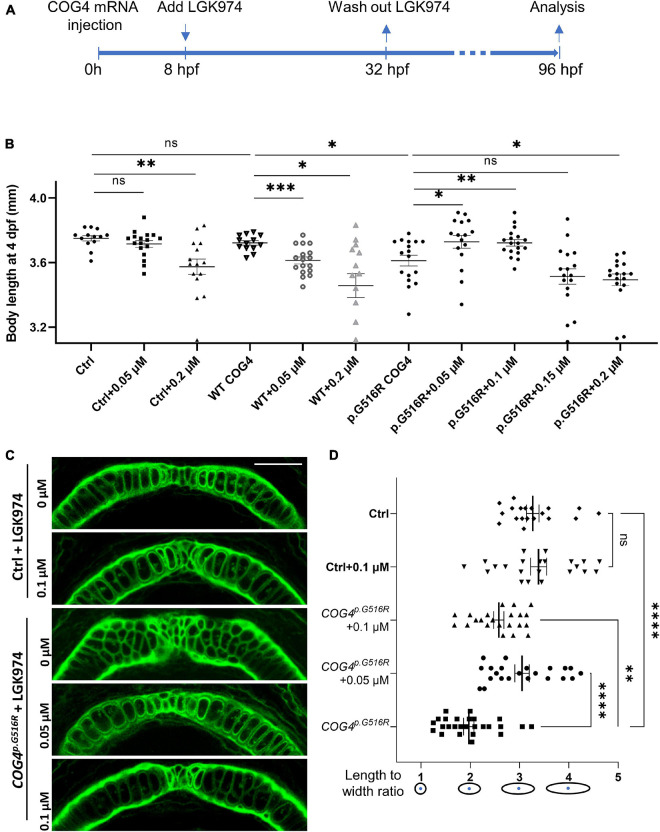
LGK974 treatment suppresses shortened body length and chondrocyte defect caused by of *COG4^*p*.G516G^* expression in zebrafish. **(A)** Scheme of the LGK974 treatment procedure. **(B)** Graphs show the measured body length of each group at 4 dpf. The data are presented as mean ± SEM. An unpaired two-tailed *t*-test was used. ^∗∗∗^*p* < 0.001; ^∗∗^*p* < 0.01; ^∗^*p* < 0.05; ns, not significant. **(C)** Ventral view of representative Meckel’s cartilage of control and *COG4^*p*.G516G^*-injected embryos with or without LGK974 treatment at 4 dpf following WGA staining and imaged by a confocal microscope. **(D)** Graphs show the length-to-width ratio of chondrocytes in Meckel’s cartilage in **(C)** and two more representative Meckel’s cartilage images of each group. The data are presented as mean ± SEM. One-way ANOVA with Tukey’s multiple comparison tests was applied. ^****^p < 0.0001; ^∗∗^*p* < 0.01; ns, not significant. Experiments were performed in two biological replicates with similar results.

We hypothesize that the increased abundance of *wnt4* contributes to the pathogenetic mechanism for SWS. Therefore, reducing Wnt4 activity may suppress the developmental defects caused by the presence of *COG4^*p*.G516R^*. LGK974 is a pharmacological inhibitor of WNT porcupine *O*-acyltransferase (PORCN), which affects palmitoylation and secretion of Wnts. After optimizing the treatment procedure (Figure 6A), we found that 0.05–0.2 μM of LGK974 significantly shortened the body length of control siblings (Supplementary Figure 5) without causing optic cup morphogenesis and shorter tail induced by high concentrations of LGK974 (Eckert et al., 2019). A 24-h incubation with low concentrations (0.05 and 0.1 μM) of LGK974 restored the shortened body length caused by *COG4^*p*.G516R^* expression at 4 dpf (Figure 6B). Higher concentrations (0.15 and 0.2 μM) of LGK974 further shortened the body length in *COG4^*p*.G516R^* injected zebrafish, showing that there is a narrow optimal range for Wnt activity. These data suggest that the imbalance of Wnt signaling may contribute to SWS pathogenesis. We also examined the chondrocyte morphology in Meckel’s cartilage and found that 0.05 and 0.1 μM of LGK974 are sufficient to suppress the deformed cartilage caused by *COG4^*p*.G516R^* expression. Up to 0.1 μM LGK974 does not cause significant defects in control chondrocytes (Figures 6C,D).

### WNT4 Is Elevated at mRNA and Protein Levels in SWS-Derived Fibroblasts

**FIGURE 7:**
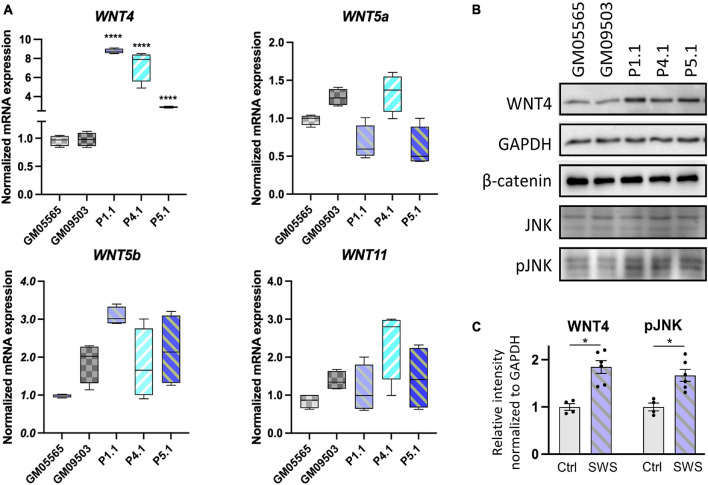
Non-canonical WNTs and related component level in SWS-derived cells. **(A)** qPCR analyses of selective non-canonical Wnt pathway components in SWS-derived fibroblasts. GAPDH was used as an internal control. The graphs represent the 2^–ΔΔCt^ values. Unpaired two-tailed *t*-test was applied for comparison of each SWS fibroblast with two controls. ^****^p < 0.0001. Experiments were performed in triplicates with similar results. **(B)** Western blotting of a few Wnt pathway components. GM05565 and GM09503 are control fibroblasts. P1.1, P4.1, and P5.1 are SWS-derived fibroblasts. **(C)** Quantitation assay of WNT4 and pJNK band density in **(B)** and the other replicates. The data are presented as mean ± SEM. An unpaired two-tailed *t*-test was used. ^∗^*p* < 0.05.

To assess whether a similar mechanism was at play in human cells, we assayed the transcript abundance of *WNT4* and other non-canonical *WNT*s in SWS individuals’ fibroblasts. Interestingly, both *WNT4* transcript and protein levels were increased in SWS individual fibroblasts (Figures 7A–C). Neither *WNT5a*, *WNT5b*, nor *WNT11* showed consistent significant differences in three SWS-derived cell lines (Figure 7A). We further detected downstream gene expression in the non-canonical pathway and found increased JNK phosphorylation (pJNK) in SWS cells compared to controls (Figures 7B,C), indicating an elevated non-canonical Wnt signaling. We further examined Wnt signaling markers in zebrafish and found that p-Rac1/cdc42, a marker for non-canonical Wnt pathway, was elevated in embryos expressing *COG4^*p*.G516R^*, but not those of *COG4^*WT*^*. A similar increase of p-Rac1/cdc42 was also seen in *wnt4* overexpression embryos (Supplementary Figure 6). β-Catenin, a marker for the canonical Wnt pathway, did not change (Figure 7B).

## Discussion

In this paper, we utilized SWS-derived fibroblasts and zebrafish, a vertebrate model to study SWS. In SWS-derived cells, the specific p.G516R amino acid substitution in COG4 selectively affected proteoglycans instead of global glycosylation. Increased glypicans were seen in both SWS-derived cells and *COG4^*p*.G516R^*-injected zebrafish embryos. Glypicans are glycosylphosphatidylinositol-anchored proteins (GPI-APs) that consist of a conserved core glycan, phosphatidylinositol, glycan side chains, and a protein moiety (Kinoshita, 2020). A recent study found that subunits of the COG complex affect proteoglycan turnover and GAG chain polymerization (Adusumalli et al., 2021). Studies also found that glycosylation changes of a cell surface protein could affect the protein’s half-life (Ohtsubo et al., 2005). Therefore, the increased glypicans are probably due to impaired protein trafficking or turnover caused by COG4^*p*.G516R^ rather than changes in glypican transcript abundance. In previous *gpc4* zebrafish studies, Topczewski demonstrated that Gpc4 regulates cellular movements during gastrulation by potentiating Wnt11f2 signaling and chondrocyte behavior independent of core Wnt pathway molecules (Topczewski et al., 2001; Sisson et al., 2015). Interestingly, low expression of *gpc4* suppressed Wnt11f2 defects, but high overexpression of *gpc4* inhibited the rescue, indicating a requirement for a fine balance between Gpc4 and Wnt11f2 to ensure normal development. This is also true for COG4 function, since more than twofold accumulation of COG4 leads to similar protein trafficking defects as seen in COG4-deficient cells (Reynders et al., 2009). Therefore, *COG4^*WT*^* is an essential control in this zebrafish study to confirm that the expression of *COG4* is not excessive to impair its normal function.

Multiple studies have shown the role of glypicans in regulating Wnt signaling. In zebrafish, Gpc4 has been reported as a positive modulator of non-canonical Wnt signaling during zebrafish gastrulation (Topczewski et al., 2001). Studies in both *Drosophila* and *Xenopus* found that glypicans can modulate the distribution of Wnt through binding the palmitoleate on Wnts and thus contribute to the release of signaling-competent Wnt (Franch-Marro et al., 2005; Yan et al., 2009; McGough et al., 2020). Among two groups of glypicans, Dally and Dally-like (Dlp) subfamilies, only Dlp has this palmitoleate-binding activity, which mainly includes Gpc4 and Gpc6 (McGough et al., 2020). Studies in chick embryos demonstrated that Gpc4 in the neural crest enhances Wnt1/Wnt3a signaling and *wnt11* expression on the dorsomedial lip (Serralbo and Marcelle, 2014). Our data also suggest the role of glypicans in regulating Wnt signaling. We hypothesize that the accumulation of glypican(s) could induce the transcriptional upregulation of *wnt4*. However, lacking a specific Gpc4 antibody in zebrafish makes it difficult to confirm whether Gpc4 is the major player in causing these phenotypes seen in *COG4^*p*.G516R^*-injected embryos. Simply overexpressing *gpc4* in zebrafish seems to show no significant defects (LeClair et al., 2009). We speculate that the abnormalities seen in *COG4^*p*.G516R^*-injected embryos are due to disrupted glypican trafficking or turnover rather than changes in glypican expression. Further studies on how glypican and Wnt4 crosstalk is yet to be demonstrated. More studies are ongoing in our lab to further address these questions. Whereas glypicans are the most impaired proteoglycans caused by the heterozygous variant in COG4 (p.G516R), we could not rule out that other proteoglycans may also contribute to the pathogenesis of SWS. Although knockdown of *dcn* and *cspg4* in zebrafish does not show chondrocyte intercalation problems, they may cause a shorter body axis or other types of cartilage malformations (Zoeller et al., 2009; Lee et al., 2020).

Besides increased glypicans, it is interesting that we also found an elevated *wnt4* transcript in SWS-derived cells and *COG4^*p*.G516R^*-injected zebrafish embryos. Studies have shown that the cell surface receptor could directly regulate WNT4 expression in chondrocytes (Dennis et al., 2020). Therefore, we hypothesize that the increased WNT4 is probably a compensatory transcriptional response to increased glypicans. Wnts are a group of secreted glycoproteins involved in cell–cell signaling (Niehrs, 2012; Willert and Nusse, 2012). To date, 22 Wnts have been identified in vertebrates (the Wnt homepage: http://web.stanford.edu/group/nusselab/cgi-bin/wnt/). Among those, Wnt4 has shown important roles in embryonic development, skeletal/bone regeneration, and sex determination with additional developmental defects in a cell-specific and tissue-specific manner (Matsui et al., 2005; Bernard and Harley, 2007; Chang et al., 2007; Rao et al., 2019; Yang et al., 2020). Most importantly, Wnt4 plays a pivotal role in regulating chondrocyte differentiation. In chick limb studies, *Wnt4* expression is found in joint cells and cartilage, and its misexpression accelerates maturation of chondrocytes, causing shortened long bones (Kawakami et al., 1999; Hartmann and Tabin, 2000; Church et al., 2002). An *in vitro* study also found that Wnt4 blocks the initiation of chondrogenesis and accelerates terminal chondrocyte differentiation (Church et al., 2002). Misexpression of *Zwnt4* (zebrafish *wnt4*) and *Xwnt4* (*Xenopus wnt4*) in zebrafish causes shortened trunk and tail (Ungar et al., 1995) and abnormal chondrocyte intercalation in Meckel’s cartilage (our data), confirming Wnt4 is involved in regulation of chondrocyte behavior, like *gpc4* and *wnt5b* (Sisson et al., 2015). In mice, Wnt4 defects led to short-limb dwarfism (Dennis et al., 2020). Most relevant, conditional expression of *Wnt4* in mice also causes dwarfism with small skeletons, dome-shaped skulls, and small jaws (Lee and Behringer, 2007). Both *Wnt4* defects and overexpression cause dwarfism, indicating Wnt4 functions within a narrow range for normal development, and bidirectional disturbance causes developmental abnormalities.

LGK974 is a PORCN inhibitor that blocks PORCN-dependent palmitoylation and secretion of WNT family ligands (Liu et al., 2013). Currently, it is being evaluated as an anticancer agent against a broad range of diseases associated with deviant Wnt signaling (Liu et al., 2013; Zhang and Lum, 2016). LGK974 has been used to inhibit canonical and non-canonical signaling in multiple studies as a WNT modifier (Han et al., 2017; Katoh, 2017; Lutze et al., 2019). In our study, titrating LGK974 allowed us to regulate the amount of Wnt during development. At low concentrations, it suppressed and nearly normalized the shortened body length caused by the presence of COG4^p.G516R^, probably by inhibiting Wnt4 (or other Wnts) palmitoylation and secretion. This rescue experiment not only supports altered Wnt signaling as one of the possible pathogenesis mechanisms contributing to SWS but also suggests a potential therapeutic strategy for SWS individuals.

In our SWS zebrafish model, the specific heterozygous dominant variant in COG4 (p.G516R) shows elevated glypicans and *wnt4*, along with developmental defects. Compared to COG4-KO zebrafish (Ferreira et al., 2018; Clement et al., 2019), it is impressive that the single mutation in COG4 is sufficient for shortened body length and abnormal chondrocyte stacking. However, there are still some differences between COG4-KO and *COG4^*p*.G516R^* zebrafish; for example, the single variant in COG4 does not decrease the overall GAG amount shown in COG4-KO zebrafish by Alcian blue staining. *COG4^*p*.G516R^* larvae also show cyclopia and a distinct pectoral fin phenotype compared to COG4-KO zebrafish.

In summary, using SWS-derived fibroblasts and a zebrafish model, we demonstrated that the specific dominant variant COG4^p.G516R^ causes the accumulation of GPI-anchored glypicans, which most likely involves COG4-dependent altered trafficking or turnover of these proteins. In zebrafish, the presence of COG4^p.G516R^ also elevates *wnt4* transcript and causes chondrocyte morphogenesis defects, which might explain the short stature and distinctive craniofacial features in SWS individuals. WNT4 and non-canonical Wnt signaling component pJNK are also elevated in cultured SWS-derived fibroblasts. We further demonstrate that the Wnt inhibitor LGK974 could suppress the defects caused by *COG4^*p*.G516R^* in zebrafish. These results from SWS-derived cell lines and zebrafish point to altered non-canonical Wnt signaling as one possible mechanism underlying SWS pathology. How increased COG4^p.G516R^ leads to elevated *WNT4* in SWS cells and zebrafish is still unknown. Transgenic and CRISPR-knock-in SWS zebrafish lines are under development to address these issues.

## Materials and Methods

### Cell Cultures

Dermal primary fibroblasts derived from healthy controls (GM08429, GM08680, GM03349, GM05565, and GM09503) were obtained from Coriell Institute for Medical Research (Camden, NJ). Each SWS-derived fibroblast line was obtained by the referring clinician and grown via a clinical laboratory service and then sent to us with consent through an approved IRB.

Fibroblasts were cultured in Dulbecco’s Modified Eagle’s medium (DMEM) containing 1 g/L glucose supplemented with 10% heat-inactivated fetal bovine serum (FBS) and 1% antibiotic–antimycotic (Life Technologies, Carlsbad, CA, United States).

### Zebrafish Husbandry

All zebrafish experiments were performed in accordance with the protocols approved by SBP IACUC. Zebrafish were maintained under standard laboratory conditions at 28.5°C. Embryos were staged according to Kimmel et al. (1995).

### Immunoblotting

For glypicans analysis, fibroblasts were treated with 5 mU/ml heparinase III in a serum-free medium for 1 h at 37°C. Zebrafish larvae were homogenized and treated with 25 mU heparinase III in H buffer (20 mM Tris-HCl, pH 7.0, 0.1 mg/ml BSA, and 4 mM CaCl_2_) for 1.5 h at 37°C. Samples were harvested using SDS lysis buffer (62.5 mM Tris-HCl, pH 6.8, 2% SDS, and 10% glycerol) supplemented with protease and phosphatase inhibitors (Sigma-Aldrich) as previously described (Ferreira et al., 2018). For analysis of PCP pathway components, 2 × 10^4^ fibroblast cells were seeded in six-well plates and harvested after 2 days using SDS lysis buffer. Equal amounts of denatured proteins were separated via SDS–polyacrylamide gel electrophoresis followed by transfer and antibody inoculation as described previously (Tambe et al., 2019). Antibodies used were Δ-heparan sulfate (AMSBIO, F69-3G10), chondroitin 6 sulfate (Millipore, MAB2035), GAPDH (Invitrogen, MA5-15738), WNT4 (R&D, MAB4751), mCherry (Rockland, 600-401-P16), COG4 (provided by Dr. Daniel Ungar, University of York, United Kingdom), β-catenin (Santa Cruz, sc-7963), JNK (sc-7345), pJNK (sc-293136), and p-Rac1/cdc42 (Cell Signaling, #2461).

### mRNA Expression Analysis

Total RNA was extracted from cells or zebrafish embryos using TRIzol^TM^ (Thermo Fisher, 15596081) reagent according to the manufacturer protocol. cDNA was synthesized using a QuantiTect Reverse Transcription Kit (QIAGEN, 205311). qPCR and data analysis were performed as described previously (Tambe et al., 2019). Briefly, primer pairs targeting genes of interest were designed using NCBI Primer-BLAST and available upon request. qPCRs were performed with the PowerUp SYBR^®^Green PCR Master Mix (Thermo Fisher, A25742). The standardized cycle conditions were applied in Applied Biosystems 7900HT Fast Real-Time PCR System. SDS2.3 software was used to analyze expression data of reference genes. The mRNA levels were normalized to the levels of housekeeping genes, *GAPDH* for fibroblasts and β*-actin* for zebrafish, and 2^–ΔΔCt^ values were calculated and compared.

### Immunofluorescence

Whole-mount immunofluorescence was performed as previously described (Alexander et al., 1998), using lectin WGA (Vector Laboratories, FL1021) against the cell membrane. Fluorescence images were acquired using an LSM 510 confocal microscope (Zeiss, Germany) with a × 40 water objective. Digital images were processed with Adobe Creative Suites.

### Cloning of Human *COG4* and *in vitro* mRNA Synthesis

Full-length human *COG4* was cloned into the pCS2 + vector from plasmid hCOG4-siR-3myc in AAZ6 (a gift from Professor Vladimir V. Lupashin) using an In-Fusion^®^ HD Cloning Kit (TaKaRa Bio, 638909) with primers 5′-ATGGGAACCAAGATGGCGGA-3′, 5′-TTACAGGCGCAG CCTCTTGATAT-3′, 5′-ATCAAGAGGCTGCGCCTGTAATCA AGGCCTCTCGAGCCTCT-3′, and 5′-TCCGCCATCTTGGTT CCCATATTCGAATCGATGGGATCCT-3′. SWS point mutation of G to A was generated using Q5^®^ Site-Directed Mutagenesis Kit (NEB, E0554S) with primers 5′-CATCCAGCGCa GGGTGACAAG-3′ and 5′-TCCTGGAAGGTGGTGGCA-3′. WT *COG4* and SWS *COG4* mRNAs (*COG4^*WT*^* and *COG4^*p*.G516R^*) were synthesized using the Invitrogen mMESSAGE mMACHINE SP6 Transcription Kit (Thermo Fisher, AM1340) following restriction enzyme *Not*I digestion and purified using a MEGAclear^TM^ Transcription Clean-Up Kit (Thermo Fisher, AM1908). One hundred picograms of mRNA was injected into each zebrafish embryo unless stated otherwise. As a complementing strategy, *Hsp70l:COG4*-*P2A-mCherry*, *cryaa:dsRED*, and SWS *COG4* were generated by cloning the coding sequence for WT *COG4* and SWS *COG4* downstream of the Hsp70l promoter in the parent vector *hsp70l:-LateSV40pA*, *cryaa:dsRED-RabBGpA*, and *I-Sce* vector (a gift from Dr. Joseph Lancman). I-*Sce*I meganuclease (NEB, R0694) was co-injected with 20 pg of the constructs per embryo.

### Cloning of Full-Length Zebrafish *wnt4* and Synthesizing Antisense Probes

Full-length zebrafish *wnt4* was cloned from cDNA into the pCS2 + vector using an In-Fusion^®^ HD Cloning Kit with the following primers: 5′-CCCATCGATTCGAATATGTCATCG GAGTATTTGATAAGGT-3′, 5′-CTCGAGAGGCCTTGATCA CCGACACGTGTGCAT-3′, 5′-TCAAGGCCTCTCGAGCCT CT-3′, and 5′-ATTCGAATCGATGGGATCCTGCA-3′. *wnt4* mRNA synthesis and purification were performed as described above. One hundred picograms of mRNA was injected into each zebrafish embryo unless mentioned otherwise. For antisense RNA probe synthesis, the full length of *wnt4* including 3′-UTR was cloned into the pGEM-T easy vector (Promega, A1360) by primers 5′-ATGTCATCGGAGTATTTGATAAGG-3′ and 5′-AGTCTTTGACACAGCATATATTTC-3′ from cDNA. After verifying the insertion direction by sequencing, the antisense RNA probe was then synthesized with SP6 RNA polymerase following *Apa*I digestion.

### Standard Whole-Mount *in situ* Hybridization

Standard whole-mount *in situ* hybridization was performed as described previously (Thisse and Thisse, 2008). INT/BCIP (175 μg/ml; Roche) was used as alkaline phosphatase substrates. The following molecular markers were used: *wnt4* and *wnt11f2* (a gift from Dr. Diane Sepich, previously used in Makita et al., 1998; Marlow et al., 2002).

### Wnt Inhibition Assay

LGK974 (Cayman Chemical, No. 14072) was dissolved in DMSO to make a 10 μM stock. Different concentrations of LGK974 were added to the control or injected zebrafish groups at 8 hpf for 24 h. DMSO of 0.01% was used as a vehicle.

## Data Availability Statement

The datasets presented in this study can be found in online repositories. The names of the repository/repositories and accession number(s) can be found below: GenBank: NM_015386.3, NM_001040387.1 (https://www.ncbi.nlm.nih.gov/nuccore/NM_015386.3).

## Ethics Statement

The animal study was reviewed and approved by the Institutional Animal Care and Use Committee (IACUC) protocols of Sanford Burnham Prebys Medical Discovery Institute.

## Author Contributions

Z-JX, X-XZ, PDSD, and HHF conceptualized the project, designed the experiments, and oversaw all studies. Z-JX, X-XZ, MT, and BGN performed the experiments and analyzed the data with HHF. Z-JX and X-XZ prepared the figures. HHF and PDSD contributed to research funding. Z-JX and X-XZ wrote the manuscript with coauthors. All authors reviewed and contributed to editing the manuscript.

## Conflict of Interest

The authors declare that the research was conducted in the absence of any commercial or financial relationships that could be construed as a potential conflict of interest.

## Publisher’s Note

All claims expressed in this article are solely those of the authors and do not necessarily represent those of their affiliated organizations, or those of the publisher, the editors and the reviewers. Any product that may be evaluated in this article, or claim that may be made by its manufacturer, is not guaranteed or endorsed by the publisher.

## Footnotes

1
www.uniprot.org

## Supplementary Material

Supplementary Figure 1 Western blots of CSPG following chondroitinase ABC digestion to generate CS-stubs. C1 and C2 are control fibroblasts. C1, GM0849; C2, GM0860. P1.1, P4.1, and P5.1 are SWS-derived fibroblasts.

Supplementary Figure 2 Expression of *COG4*^*p.G516R*^ in zebrafish shows abnormal chondrocyte intercalation of Meckel’s cartilage at the late developmental stage. Ventral view of representative Meckel’s cartilage of zebrafish larvae at 7 dpf after Alcian blue staining, imaged by a light dissecting microscope. Dotted circular lines highlight cell shape and their relative configuration with each other.

Supplementary Figure 3 Expression of *COG4*^*p.G516R*^ in zebrafish causes cyclopia, stunted fin, and abnormal ceratohyal cartilage in zebrafish. **(A)** Expression of *COG4*^*p.G516G*^ causes cyclopia in zebrafish. Dorsal view of representative images of cyclopia compared to control and *COG4*^*WT*^. Two hundred picograms of *COG4*^*WT*^ or *COG4*^*p.G516G*^ mRNA was injected into each embryo. **(B)** Ventral view of representative zebrafish larvae at 6 dpf after Alcian blue staining, imaged by a light dissecting microscope. Twenty picograms of DNA was injected into each embryo, and heat shock was performed at 24 and 46 hpf for 1 h each at 38°C.

Supplementary Figure 4 *wnt4* upregulation shows a dose-dependent response to *COG4*^*p.G516R*^ mRNA. Bar graphs represent quantitative PCR analyses of *wnt4* at 6 hpf after injection of different amounts of *COG4*^*p.G516R*^ mRNA. *wnt4* expression was normalized to β*-actin*. ^****^*p* < 0.0001; ^∗∗^*p* < 0.01; ns, not significant.

Supplementary Figure 5 Dose-dependent response analysis of the Wnt inhibitor LGK974 in zebrafish. **(A)** Body length at 3 dpf after LGK974 treatment. **(B)** Representative images after LGK974 treatment at 3 dpf. The data are presented as mean. ^****^*p* < 0.0001.

Supplementary Figure 6 Expression of *COG4*^*p.G516R*^ and *wnt4* elevated non-canonical Wnt signaling in zebrafish. Samples for Western blots were collected at 6 hpf. The p-Rac1/cdc42 level increased in embryos injected with *COG4*^*p.G516R*^ mRNA, but not with *COG4*^*WT*^ mRNA. Similar elevation of p-Rac1/cdc42 was also seen in *wnt4* overexpression embryos. β-Catenin did not change.
