# Supplementary figures and images for: A Dominant Heterozygous Mutation in COG4 Causes Saul–Wilson Syndrome, a Primordial Dwarfism, and Disrupts Zebrafish Development via Wnt Signaling
Source: Front Cell Dev Biol. 2021 Sep 14;9:720688. doi: 10.3389/fcell.2021.720688 (PMC8476873; doi:10.3389/fcell.2021.720688)

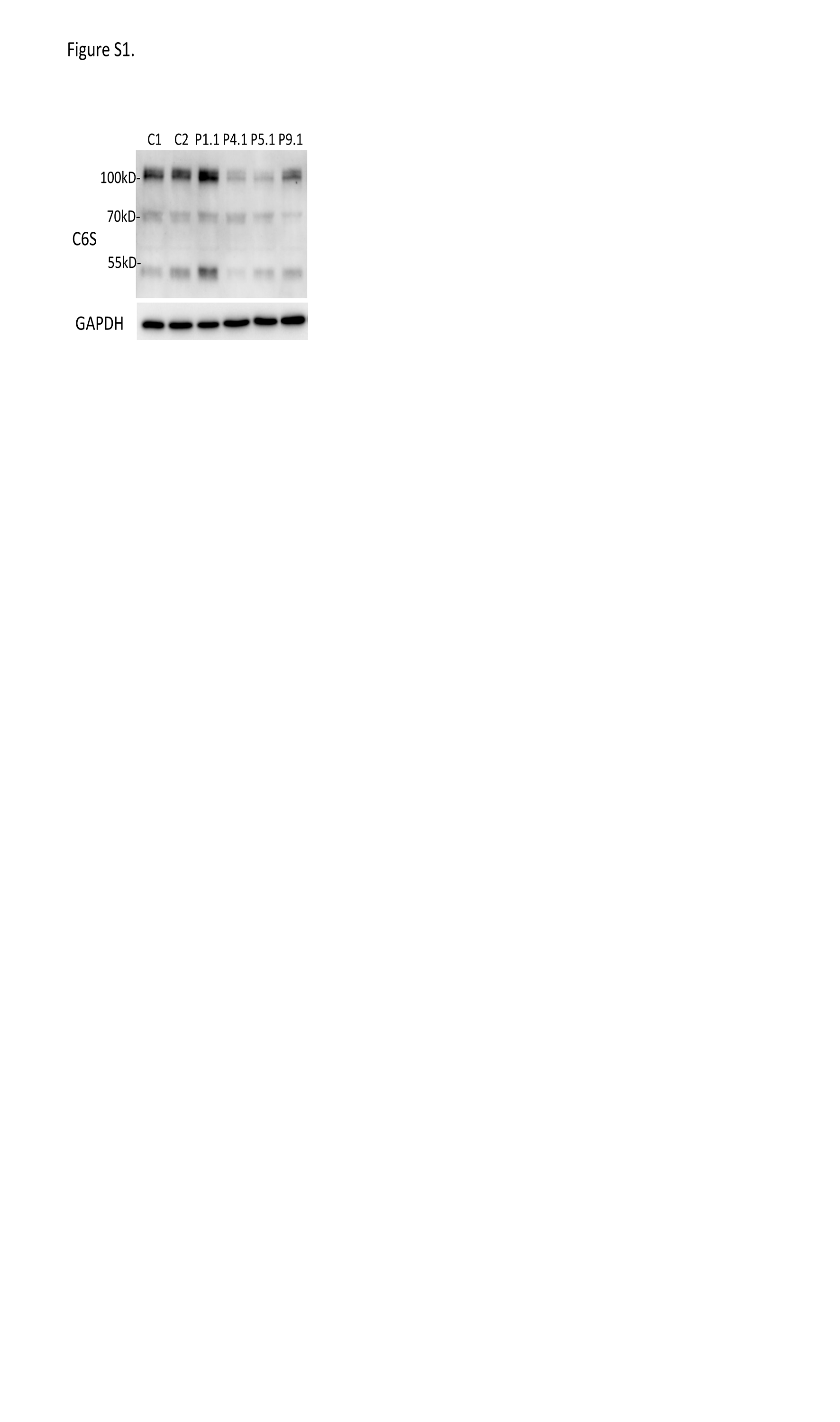

Supplement: Supplementary Figure 1 — Western blots of CSPG following chondroitinase ABC digestion to generate CS-stubs. C1 and C2 are control fibroblasts. C1, GM0849; C2, GM0860. P1.1, P4.1, and P5.1 are SWS-derived fibroblasts. [file Image_1.tif]

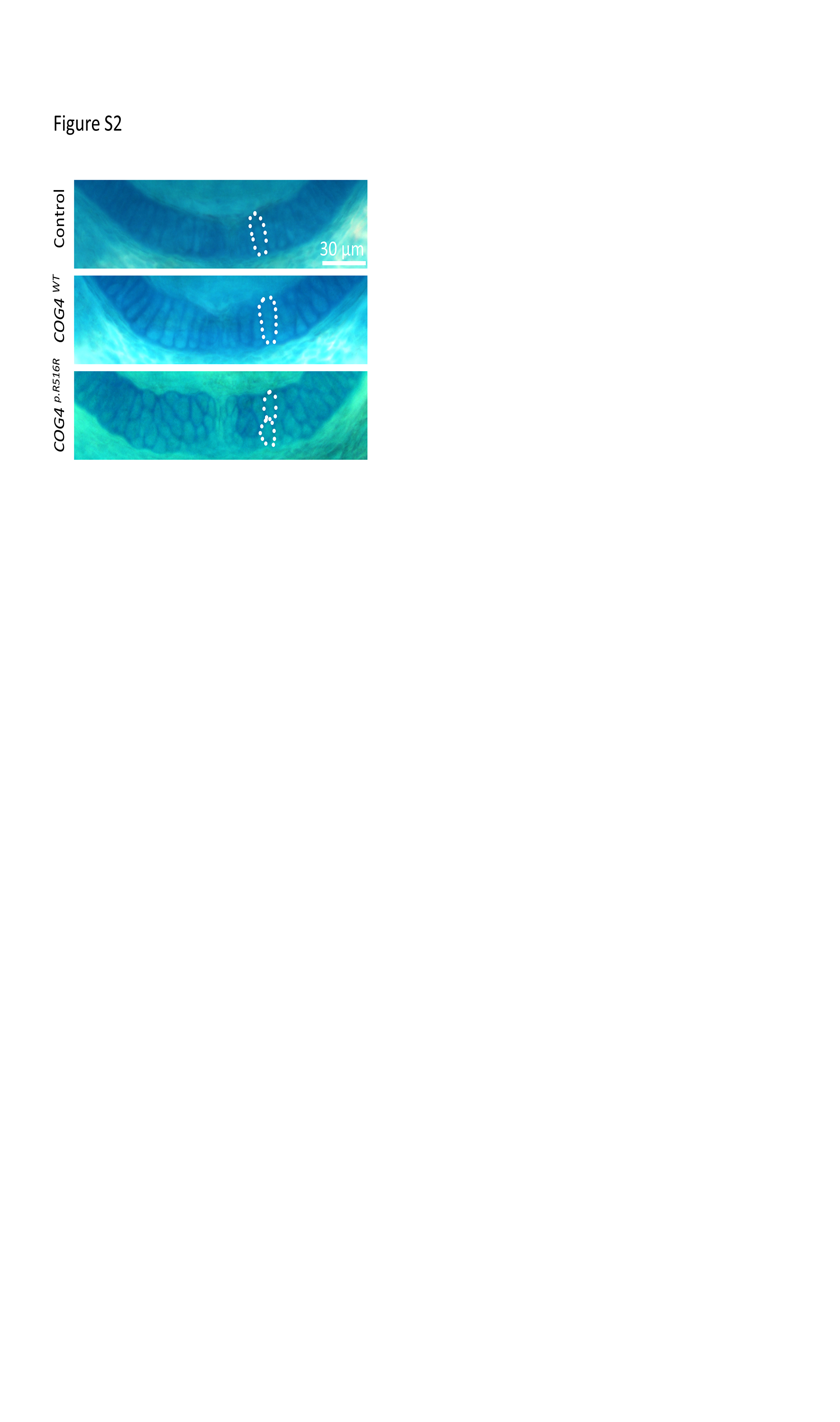

Supplement: Supplementary Figure 2 — Expression of COG4p.G516R in zebrafish shows abnormal chondrocyte intercalation of Meckel’s cartilage at the late developmental stage. Ventral view of representative Meckel’s cartilage of zebrafish larvae at 7 dpf after Alcian blue staining, imaged by a light dissecting microscope. Dotted circular lines highlight cell shape and their relative configuration with each other. [file Image_2.tif]

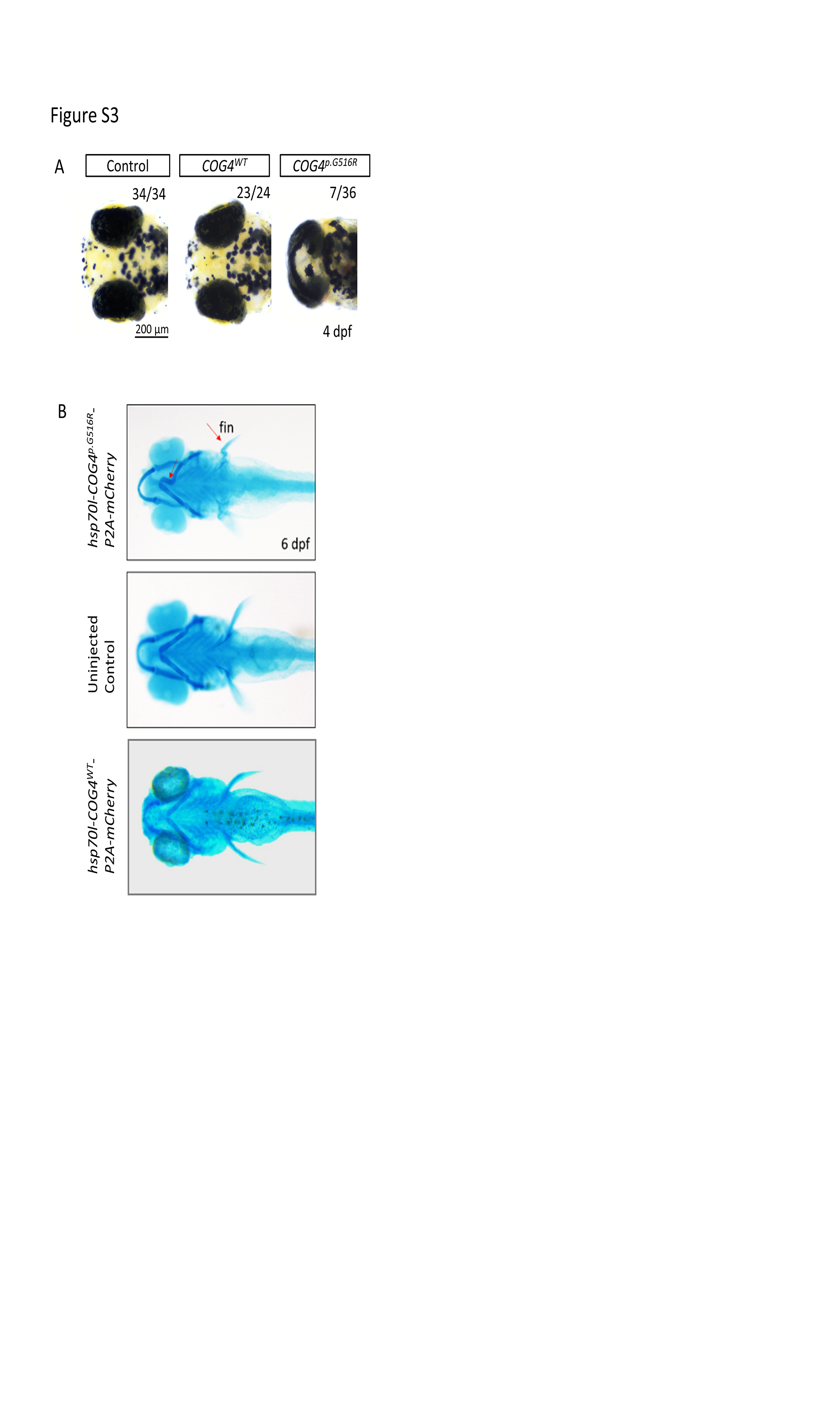

Supplement: Supplementary Figure 3 — Expression of COG4p.G516R in zebrafish causes cyclopia, stunted fin, and abnormal ceratohyal cartilage in zebrafish. (A) Expression of COG4p.G516G causes cyclopia in zebrafish. Dorsal view of representative images of cyclopia compared to control and COG4WT. Two hundred picograms of COG4WT or COG4p.G516G mRNA was injected into each embryo. (B) Ventral view of representative zebrafish larvae at 6 dpf after Alcian blue staining, imaged by a light dissecting microscope. Twenty picograms of DNA was injected into each embryo, and heat shock was performed at 24 and 46 hpf for 1 h each at 38°C. [file Image_3.tif]

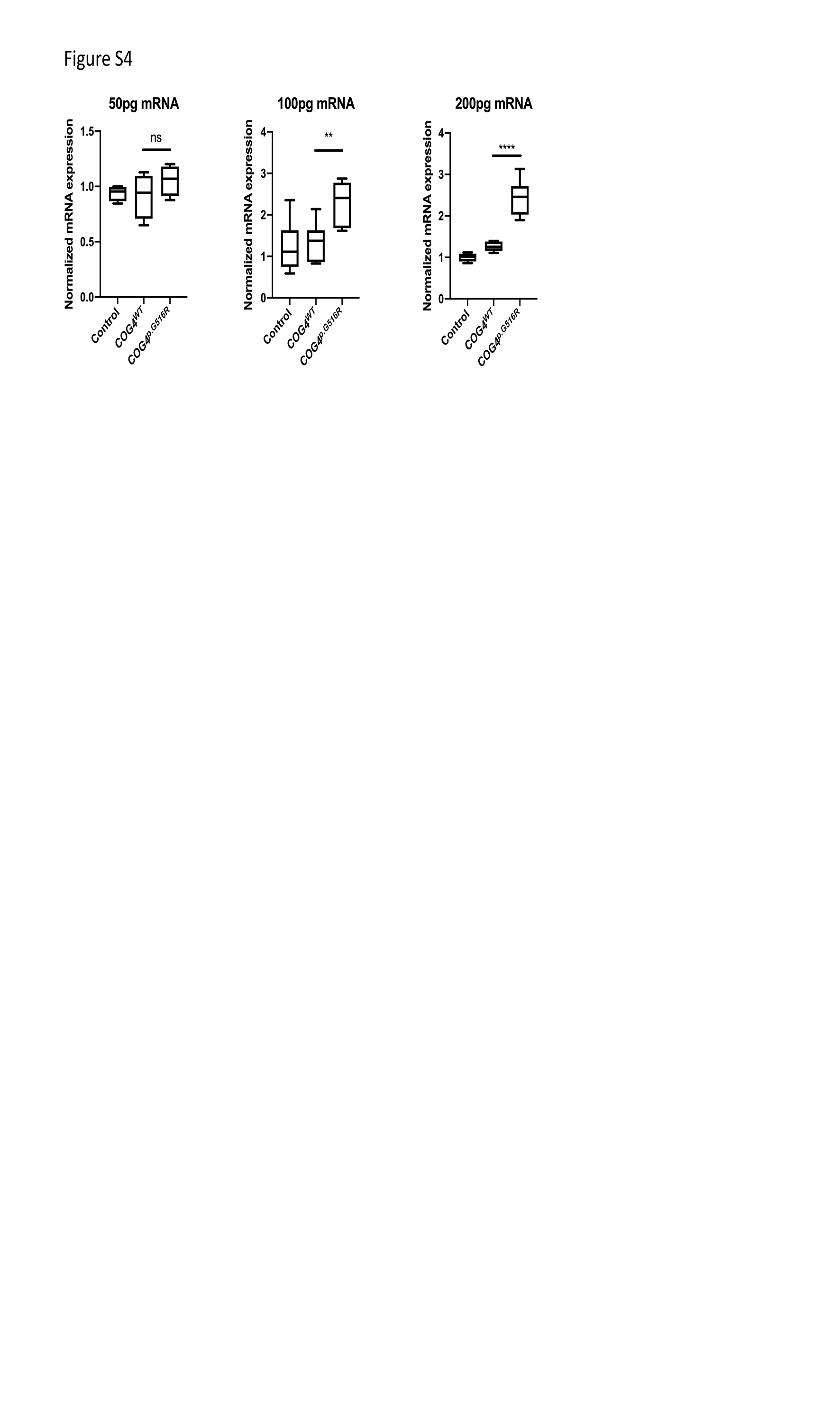

Supplement: Supplementary Figure 4 — wnt4 upregulation shows a dose-dependent response to COG4p.G516R mRNA. Bar graphs represent quantitative PCR analyses of wnt4 at 6 hpf after injection of different amounts of COG4p.G516R mRNA. wnt4 expression was normalized to β-actin. ****p < 0.0001; ∗∗p < 0.01; ns, not significant. [file Image_4.tif]

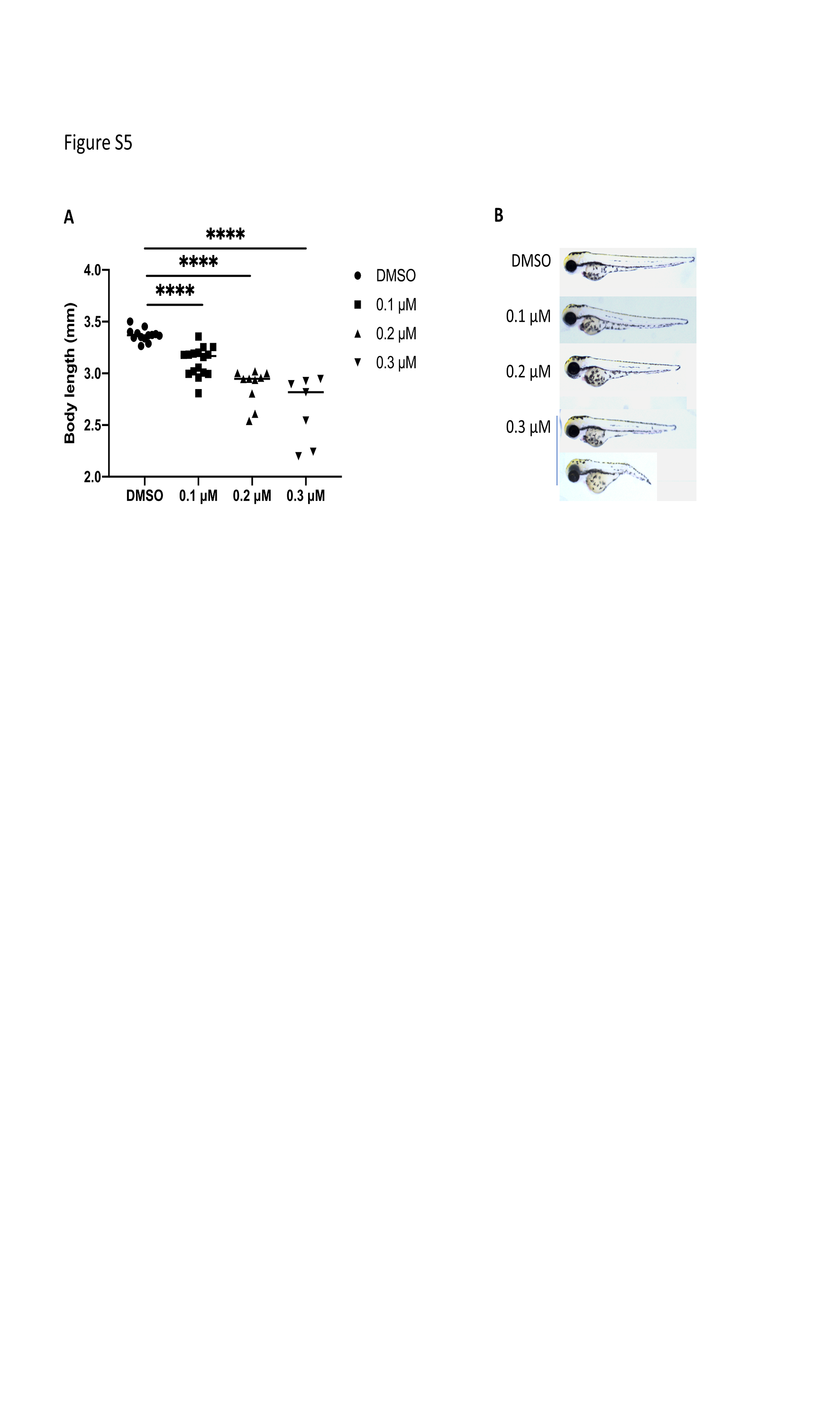

Supplement: Supplementary Figure 5 — Dose-dependent response analysis of the Wnt inhibitor LGK974 in zebrafish. (A) Body length at 3 dpf after LGK974 treatment. (B) Representative images after LGK974 treatment at 3 dpf. The data are presented as mean. ****p < 0.0001. [file Image_5.tif]

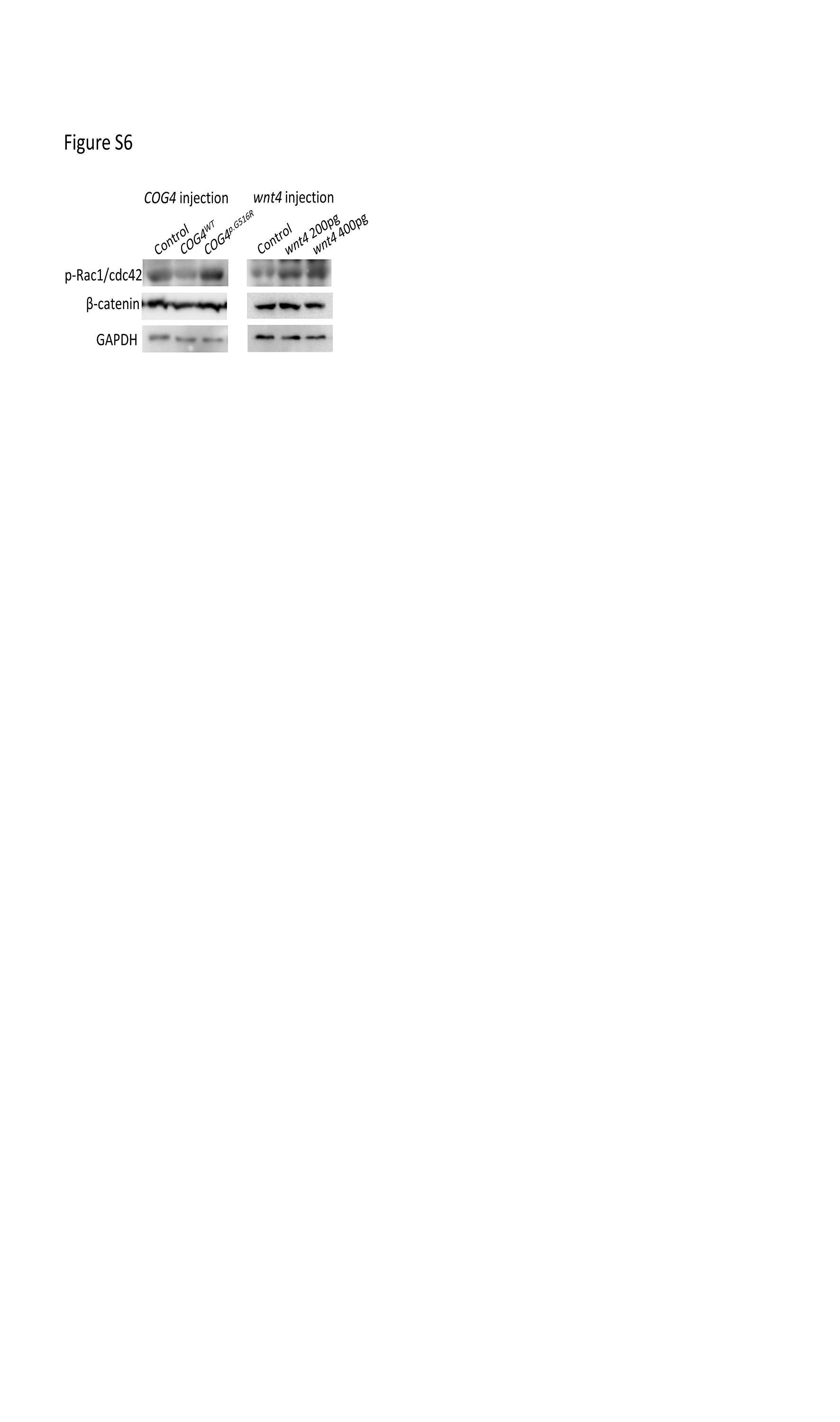

Supplement: Supplementary Figure 6 — Expression of COG4p.G516R and wnt4 elevated non-canonical Wnt signaling in zebrafish. Samples for Western blots were collected at 6 hpf. The p-Rac1/cdc42 level increased in embryos injected with COG4p.G516R mRNA, but not with COG4WT mRNA. Similar elevation of p-Rac1/cdc42 was also seen in wnt4 overexpression embryos. β-Catenin did not change. [file Image_6.tif]
